# Early Diagnosis of Pneumonia and Chronic Obstructive Pulmonary Disease with a Smart Stethoscope with Cloud Server-Embedded Machine Learning in the Post-COVID-19 Era

**DOI:** 10.3390/biomedicines13020354

**Published:** 2025-02-04

**Authors:** Direk Sueaseenak, Peeravit Boonsat, Suchada Tantisatirapong, Petcharat Rujipong, Sirapat Tulatamakit, Onanong Phokaewvarangkul

**Affiliations:** 1 Department of Biomedical Engineering, Faculty of Engineering, Srinakharinwirot University, Nakhon Nayok 26120, Thailand; direks@g.swu.ac.th (D.S.);; 2 Department of Adult and Gerontological Nursing, Faculty of Nursing, Srinakharinwirot University, Nakhon Nayok 26120, Thailand; 3 Department of Medicine, Faculty of Medicine, Srinakharinwirot University, Nakhon Nayok 26120, Thailand; 4 Department of Medicine, Faculty of Medicine, Chulalongkorn University, Bangkok 10330, Thailand

**Keywords:** respiratory sound, respiratory disease, pneumonia, chronic obstructive pulmonary disease, machine learning, mobile application

## Abstract

**Background/Objectives**: Respiratory diseases are common and result in high mortality, especially in the elderly, with pneumonia and chronic obstructive pulmonary disease (COPD). Auscultation of lung sounds using a stethoscope is a crucial method for diagnosis, but it may require specialized training and the involvement of pulmonologists. This study aims to assist medical professionals who are non-pulmonologist doctors in early screening for pneumonia and COPD by developing a smart stethoscope with cloud server-embedded machine learning to diagnose lung sounds. **Methods**: The smart stethoscope was developed using a Micro-Electro-Mechanical system (MEMS) microphone to record lung sounds in the mobile application and then send them wirelessly to a cloud server for real-time machine learning classification. **Results**: The model of the smart stethoscope classifies lung sounds into four categories: normal, pneumonia, COPD, and other respiratory diseases. It achieved an accuracy of 89%, a sensitivity of 89.75%, and a specificity of 95%. In addition, testing with healthy volunteers yielded an accuracy of 80% in distinguishing normal and diseased lungs. Moreover, the performance comparison between the smart stethoscope and two commercial auscultation stethoscopes showed comparable sound quality and loudness results. **Conclusions**: The smart stethoscope holds great promise for improving healthcare delivery in the post-COVID-19 era, offering the probability of the most likely respiratory conditions for early diagnosis of pneumonia, COPD, and other respiratory diseases. Its user-friendly design and machine learning capabilities provide a valuable resource for non-pulmonologist doctors by delivering timely, evidence-based diagnoses, aiding treatment decisions, and paving the way for more accessible respiratory care.

## 1. Introduction

Respiratory diseases encompass a wide range of disorders affecting the lungs and related structures, including the airways, parenchyma, and pulmonary vasculature involved in the overall function of breathing [1]. These conditions impair respiratory function, leading to distressing symptoms such as dyspnea (shortness of breath) and chronic cough, which can significantly impact a patient’s quality of life physically, mentally, socially, and economically [2,3]. Of the 41 million annual deaths attributed to non-communicable diseases worldwide, 4.1 million are caused by respiratory disease, highlighting their importance in healthcare management [4,5,6]. Notably, the prevalence of respiratory disease increases with age, contributing to significant morbidity and mortality in older populations [3]. Among elderly individuals, pneumonia and chronic obstructive pulmonary disease (COPD) are common, presenting with varied symptoms that can pose diagnostic challenges for non-pulmonologist doctors, which is a major concern during pandemics such as COVID-19 [7,8,9]. As a result, early detection of specific lung abnormalities through a stethoscope is vital, as it enables accurate diagnosis and timely treatment. However, diagnosing and treating these conditions can be complex, often requiring healthcare providers to be well-trained in stethoscope use and possess in-depth knowledge of respiratory diseases [10]. In many cases, management should be aligned with clinical practice guidelines, and the involvement of a pulmonologist is necessary to ensure accurate diagnosis and optimal treatment options [11].

The use of a stethoscope to evaluate lung pathology involves auscultating various areas of the chest to detect abnormal lung sounds, which can help identify specific respiratory conditions [10]. To assess lung function, a doctor will typically listen to the front, back, and sides of the chest, focusing on both inhalation and exhalation sounds. These sounds are generated by airflow through the respiratory tract and are categorized into two groups: breath sounds and adventitious sounds [10]. Normal breath sounds include bronchial, bronchovesicular, and vesicular sounds, which are specific to their anatomical locations. Bronchial sounds are produced by airflow through the trachea and are typically heard at the sternoclavicular joint. Vesicular sounds are generated by airflow in the alveoli and are listened to over the lung area. Bronchovesicular sounds arise from airflow in the bronchus and are audible in the upper third of the anterior chest [12]. Adventitious sounds indicate pathology, including crackles, wheezes, rhonchi, and stridor. Wheezing results from the fluttering of narrowed airways and is associated with airway obstruction [12,13]. Rhonchi sounds are produced by airflow in constricted large airways and are often related to conditions like asthma. Crackles are generated by airflow through constricted airways and are commonly heard in patients with pneumonia or COPD [12,13]. Stridor is a high-pitched sound originating from the upper airway due to obstruction or swelling. Additionally, the volume of audible breath sounds can indicate conditions such as pleural effusion or lung consolidation [12].

Pneumonia often produces localized crackles or rales, which are caused by fluid in the alveoli and small airways, resembling the sound of hair being rubbed between fingers [12]. These crackles are typically heard in a specific area of the lungs where the infection is concentrated. In contrast, COPD usually presents with more diffuse and bilateral wheezing or rhonchi, caused by narrowed airways and mucus obstruction. Wheezing is a high-pitched, whistling sound, while rhonchi are deeper, resembling snoring [12]. Properly distinguishing between these sounds can aid in early and accurate diagnosis, guiding appropriate treatment for elderly patients [14]. However, in elderly patients, differentiating between the lung sounds of pneumonia and COPD can be both crucial and challenging [15]. This is because both conditions may occur simultaneously, as community-acquired pneumonia is common in hospitalized patients and may lead to an acute exacerbation of COPD [16]. Moreover, COVID-19 infection can exacerbate COPD or lead to COVID-19 pneumonia [17]. Therefore, accurate diagnosis by distinguishing between these diseases is critically important in these difficult cases. However, most pulmonologists are based in large referral hospitals, making it difficult for all suspected patients to access appropriate medical consultation, especially those in rural areas or during events like the COVID-19 pandemic with lockdown situations [18].

Artificial intelligence (AI) is becoming increasingly popular nowadays as a tool to support and augment human work [19]. AI also offers a promising solution for improving lung auscultations by enabling remote diagnosis and differentiation between various respiratory conditions. Machine learning (ML) is a subset of AI that automatically enables a system to learn and train to an algorithm once it has been exposed to more training datasets [20]. This approach enables more efficient and accurate diagnosis, helping healthcare professionals manage large volumes of data and improve decision-making processes in various fields, including medicine, especially respiratory medicine [19]. Nowadays, chest X-ray images are widely used to identify respiratory conditions [21,22], but this still requires a radiologist or a clinician to collect and analyze large amounts of data over an extended period. Moreover, AI has been applied to medical imaging techniques for improving the diagnostic efficiency of pulmonologists in differentiating COVID-19 pneumonia from community-acquired pneumonia by using CT scans, with promising results [23], or for prognostication purposes in patients with COVID-19 by using chest X-ray data [24]. In addition, several studies have been conducted on the use of auscultation sounds for diagnosis, such as coughing for the diagnosis of coronavirus [25], breathing for the diagnosis of several respiratory diseases, including COPD [26,27,28,29], and respiratory sounds for the diagnosis of several lung diseases [27,28,30,31]. Various machine learning techniques, such as wavelet transform, statistical values, and ensemble classifiers, have been applied for these purposes [27,32]. While previous studies have reported high diagnostic efficacy, they focused on classifying only a single disease at a time, even though patients can often experience more than one respiratory disorder simultaneously. Therefore, integrating telemedicine and AI offers a promising solution for enhancing healthcare access and quality with remote monitoring and consultation among patients with respiratory conditions like COPD and pneumonia [33,34]. This issue underscores the need for developing a self-recording stethoscope with AI algorithm-assisted auscultation to distinguish respiratory diseases [35]. Such a tool would greatly benefit non-pulmonologist doctors by providing faster, more accurate diagnoses and improving treatment decisions. This approach not only bridges the gap in healthcare accessibility but also enhances patient care for patients residing in rural areas or during situations like pandemics or when travel is restricted.

Therefore, this study aims to develop an application-based smart stethoscope for screening respiratory disease, capable of reporting the probabilistic likelihood of one of four conditions: healthy individuals, COPD, pneumonia, or other respiratory diseases. Moreover, non-MD medical professionals can also use the system as an out-of-hospital tool to detect common respiratory diseases early. Ultimately, the system will help to advise individuals who are suspected of having respiratory diseases to consult pulmonologists at larger hospitals, if necessary. Moreover, individuals’ personal health information will be securely stored after their providing consent, and their data will be secured without being shared with any third party without the patients’ explicit consent. To ensure privacy and prevent the identification of data in a cloud-based server, the cloud will securely encrypt and anonymize these data. Additionally, we will encrypt username and password information at every stage of cross-platform data transmission during login or registration to ensure security. However, if individuals consent to sharing their health information, the system can potentially be integrated with telemedicine by transmitting the recorded lung sounds for additional consultation. This will allow pulmonologists at a specialized center to confirm the diagnosis and recommend treatment for patients remotely.

## 2. Materials and Methods

**Figure 1:**
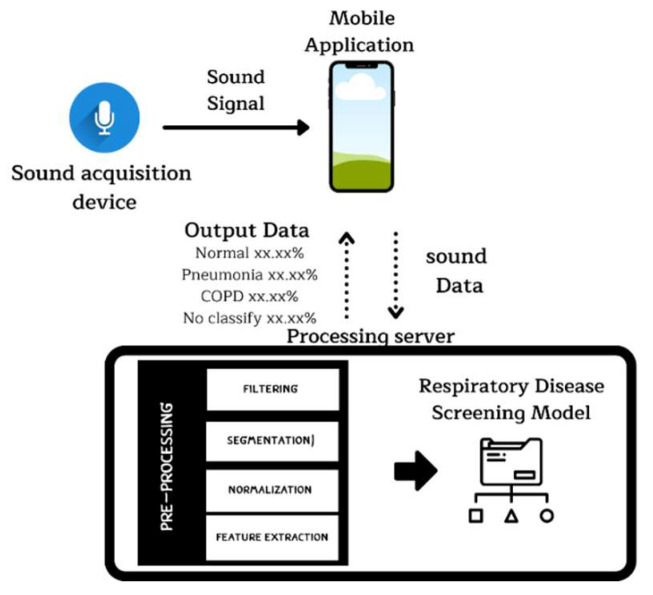
The workflow of the proposed respiratory screening system.

The development of the smart stethoscope involves three main components, as illustrated in Figure 1: (1). development of the smart stethoscope, (2) development of a mobile application with a processing server, and (3). development of an embedded AI algorithm with a respiratory disease screening model in the cloud for real-time analysis.

### 2.1. Development of the Smart Stethoscope

The first step focuses on creating a smart stethoscope equipped with a sound acquisition device. This device is designed to capture high-quality lung and respiratory sounds from patients and store information in a mobile application and then transmit the data wirelessly to a server for further processing. The smart stethoscope is lightweight, portable, and user-friendly, which allows healthcare providers or patients to easily use it outside of hospital settings. The design of the respiratory sound acquisition system captures both respiratory and cardiac sounds from the chest and then converts them into digital signals to be processed on a respiratory disease classification server. The process begins with the sound acquisition device, which consists of a stethoscope diaphragm, a Micro-Electro-Mechanical system (MEMS) audio sensor with an omnidirectional digital microphone on the sound module, a sound cone case, and a sealed case. The device is responsible for converting audio signals into digital signals, which are then transmitted via cables to mobile applications. The digital microphones output a stable digital signal resistant to interference [36].

This study uses the BlueCoin Starter Kit (STMicroelectronics) to capture respiratory sounds. The BlueCoin Starter Kit is an integrated development and prototyping platform for augmented acoustic and motion sensing in IoT (Internet of Things) applications. When the respiratory sound signal passes through the diaphragm to the BlueCoin board, which is equipped with a high-sensitivity 4-digital Micro-Electro-Mechanical System (MEMS) microphone, the captured sound signal is converted into a digital format and transmitted to the microcontroller. The data are then sent to the digital-to-audio converter, which converts the digital data back into analog data. The respiratory sound signal is subsequently transmitted to a smartphone application via a 3.5 mm connector. Regarding the frequency response of the sound capture system, a normal feature of sound conduction in human lungs lies within the 80–1000 Hz frequency range [37]. In the smart stethoscope, the interference noise is filtered using a 5th-order Butterworth bandpass filter which combines a low-pass filter with a cutoff frequency of 1000 Hz and a high-pass filter with a cutoff frequency of 80 Hz. This filter is embedded on the server. The system’s signal-to-noise ratio (SNR) is 36.17 dB. Therefore, the smart stethoscope can accurately detect sound frequencies in the 80 to 1000 Hz range. A decrease in the power spectrum was observed when the sound frequency exceeded 1000 Hz, reflecting the noise cancellation capability of our smart stethoscope.

### 2.2. Development of Mobile Application with a Processing Server

The second step involves developing the mobile application, created using Android Studio, which acts as an intermediary between the device and the server. It uses wireless signals with Transmission Control Protocol/Internet Protocol (TCP/IP) to send audio data from the device to the server and retrieves the processing results from the server to display on the mobile application interface. The server is developed using the Flask web framework, which hosts the respiratory screening model and data pre-processing program. The data preparation program includes digital filtering, normalization, segmentation, and feature extraction, all implemented using Python.

### 2.3. Development of Machine Learning Model Embedded into a Cloud-Based Respiratory Disease Screening System for Real-Time Analysis

**Figure 2:**
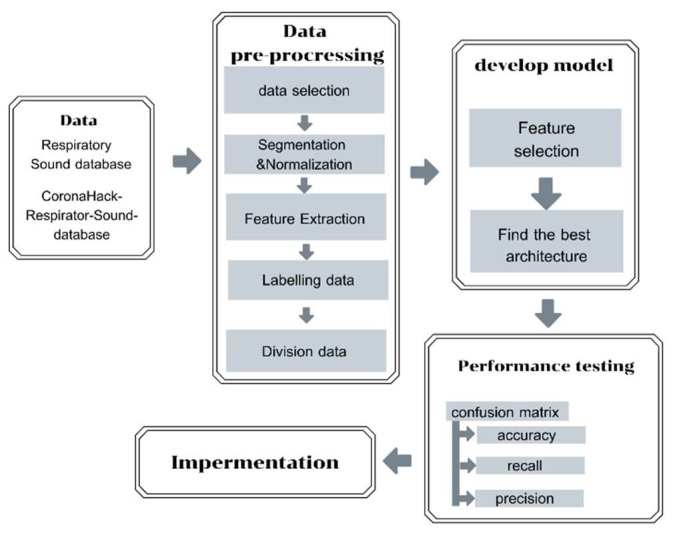
Model development and evaluation procedures.

The third step involves embedding a machine learning (ML) model into a cloud-based system. Once the auscultation sounds are transmitted from the smart stethoscope and sent to the cloud-based system, it will analyze the data using a pre-trained ML model developed in Python. This model can classify the sounds into one of four categories: healthy, COPD, pneumonia, or other respiratory diseases. The ML model provides a probabilistic diagnosis based on the sound patterns it detects, and the results are displayed on a mobile application in real time. This approach allows for quick and accurate screening, aiding in early detection and timely intervention, even in out-of-hospital settings. Figure 2 represents the process of developing and evaluating the model’s performance, which includes data collection, data pre-processing, development and evaluation of the model, performance testing, and model implementation. More details on the five-step development process are provided below.

#### 2.3.1. Data Collection

We combined data from the respiratory sound database and CoronaHack respiratory sound dataset to increase the number of respiratory sound data points for patients with coronavirus disease. We now have respiratory sounds of patients with various respiratory diseases, including lower respiratory tract infection (LRTI), upper respiratory tract infection (URTI), asthma, COPD, pneumonia, bronchiolitis, bronchiectasis, and coronavirus disease. These sounds are categorized as follows: 1397 for coronavirus disease, 2 for lower respiratory tract infections (LRTI), 14 for upper respiratory tract infections (URTI), 26 for healthy individuals, 1 for asthma, 64 for COPD, 6 for bronchiolitis, 7 for bronchiectasis, and 6 for pneumonia (https://www.kaggle.com/datasets/praveengovi/coronahack-respiratory-sound-dataset, date of data retrieval: 30 September 2021). In this study, we classify the data into four conditions: healthy individuals, pneumonia, COPD, and other respiratory diseases (including coronavirus disease, asthma, LRTI, URTI, bronchiolitis, and bronchiectasis) to create a first-generation model.

#### 2.3.2. Pre-Processing

In general, respiratory sounds have frequencies between 80 and 1000 Hz [13,37], so bandpass filters must have a bandpass within that range. We are interested in using digital filters because digital signal processing involves mathematical operations for signal tuning [38]. In this study, we divided the respiratory sound dataset into training and testing datasets at a ratio of 80:20, respectively. Then, we filtered the respiratory sound signals using four types of digital filters, including Butterworth, Chebyshev Type 1, Chebyshev Type 2, and Elliptic, to observe their frequency responses. These filters also compared the power spectrum to the commercial stethoscope.

#### 2.3.3. Feature Extraction

From the literature review, the optimal sound length for analyzing respiratory sounds is 6 s. Due to a mother wavelet, which is a mathematical function of a small wave current that represents translated and scaled copied of a finite-length waveform, the optimal mother wavelet is a Daubechies wavelet (db7) [39]. Using the statistical data, a 10th-level wavelet-transformed signal was found to improve the model’s efficiency [32]. Additionally, the entropy feature can boost the model’s accuracy up to 90% [27]. Therefore, we are interested in the entropy feature (Shannon entropy and spectral entropy) and statistical data (root mean square) of the frequency component coefficient from the discrete wavelet transform signal. In this study, we normalize the measured signals to simplify them and ensure that the values fall within the range of [−1, 1]. Then, using the fixed-length segment method, the measured signals are segmented into 6-s intervals, covering the duration of a breathing cycle. Therefore, wavelet transformation using the Daubechies wavelets (db7) of order 10 was applied to selected data. Finally, the measured signals undergo feature extraction using Shannon entropy and spectral entropy, and statistical data undergo feature extraction with the root mean square (RMS) of the frequency component coefficient from the discrete wavelet transform signal. For the spectral entropy method, this is calculated by analyzing the signal to determine its energy in the frequency domain. We compute the probability distribution of energy across each frequency band using the energy data. For the Shannon entropy method, this is calculated by using log2; this method calculates entropy over the entire signal segment, specifically within a 6-s duration.

**Figure 3:**
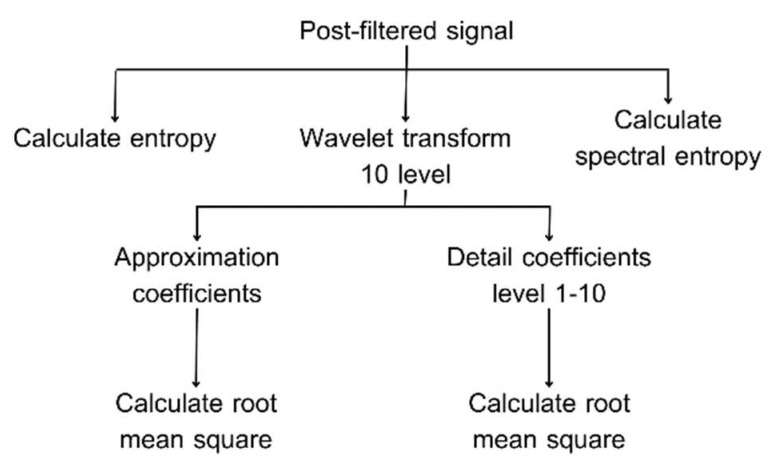
The feature extraction process.

The process is displayed in Figure 3.

Shannon entropy, spectral entropy, and root mean square are presented in Equations (1)–(3).

(1) $$\mathrm{Shannon}\;\mathrm{Entropy}=-\sum_{i=1}^{n}p\left(i\right)\log_{2}p\left(i\right)$$

p(i) is the probability that the data value will be i.

(2) $$\mathrm{Spectral}\;\mathrm{entropy}=-\sum_{f=0}^{\frac{f_{s}}{2}}P(f)\log_{2}\left[P(f)\right]$$

P(f) is the power spectral density of that frequency.

(3) $$\mathrm{Root}\;\mathrm{Mean}\;\mathrm{Square}=\sqrt{\frac{1}{N}\sum_{i=1}^{N}{D_{i}}^{2}}$$

D_i_ is the value of the i-order frequency component.

The feature extraction results are subsequently selected by the Kruskal–Wallis statistical test or H-test. This is a one-way nonparametric test that tests the differences between more than two independent groups of data by ranking feature values. It is shown in Equation (4) [40]

(4) $$H=\frac{12}{N\left(N+1\right)}\sum_{i=1}^{k}\frac{R_{i}^{2}}{n_{i}}-3\left(N+1\right)$$

where H is Kruskal–Wallis statistical, N is the number of total data, n_i_ is the number of data class i, and R_i_ is the sum of ranks in group i data.

The H-value is compared with the critical value obtained from the chi-squared distribution table at a degree of freedom of *k* − 1, where *k* is the number of groups. If the value from the table is greater than the H-value, we accept that the data for each group are not different. However, if the value from the table is less than the H-value, we reject the hypothesis that the groups of data are the same.

#### 2.3.4. Classification

This study used the artificial neural network (ANN) technique. ANNs are simulations of neural networks that consist of an input layer, a hidden layer, and an output layer. Each layer contains nodes or perceptrons. In the input layer, the number of nodes corresponds to the number of input data appointed, while in the output layer, the number of nodes corresponds to the number of classified classes. Developers can set the number of nodes in the hidden layer and adjust functions in the model [20,41]. At this stage, we chose to study a model to classify three diseases: pneumonia, COPD, and other respiratory diseases. We selected the rectified linear activation function in the hidden layer, the softmax activation function in the output layer, and the categorical cross-entropy function as the model’s loss function. The process of optimizing the model included dropping out layers to avoid overfitting, adding a regularization term to the cost function to make it more general, and starting training with early stopping for the model.

#### 2.3.5. Performance Testing

For performance testing, Python software version 3.10 was used to analyze the model’s performance. After implementing the classification model, the dataset from the database and volunteers was used to evaluate the system’s efficiency. The confusion matrix was computed to quantify the effectiveness of the system in terms of specificity, sensitivity, precision, and accuracy, as explained in Equations (5)–(8)

(5) $$\mathrm{specificity}=\frac{T_{n}}{T_{n}{+F}_{p}}$$

(6) $$\mathrm{sensitivity}=\frac{T_{p}}{T_{p}{+F}_{n}}$$

(7) $$\mathrm{precision}\;=\frac{T_{p}}{T_{p}+F_{p}}$$

(8) $$\mathrm{accuracy}=\frac{T_{p}+T_{n}}{T_{p}+T_{n}+F_{p}+F_{n}}$$

where T_n_ is true negative, T_p_ is true positive, F_p_ is false positive, and F_n_ is false negative.

## 3. Results

### 3.1. Results of the Smart Stethoscope: Design and Performance Tests

#### 3.1.1. Design of the Smart Stethoscope

**Figure 4:**
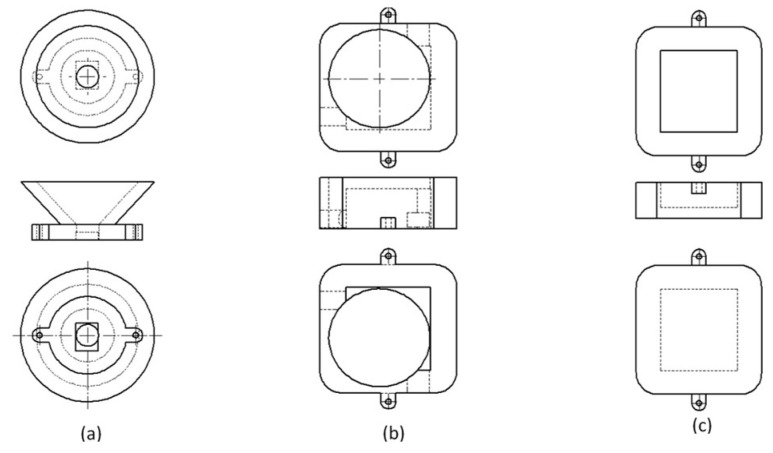
Design of the smart stethoscope showing (**a**) the sound cone, (**b**) the top part of the sealed case, and (**c**) the bottom part of the sealed case.

**Figure 5:**
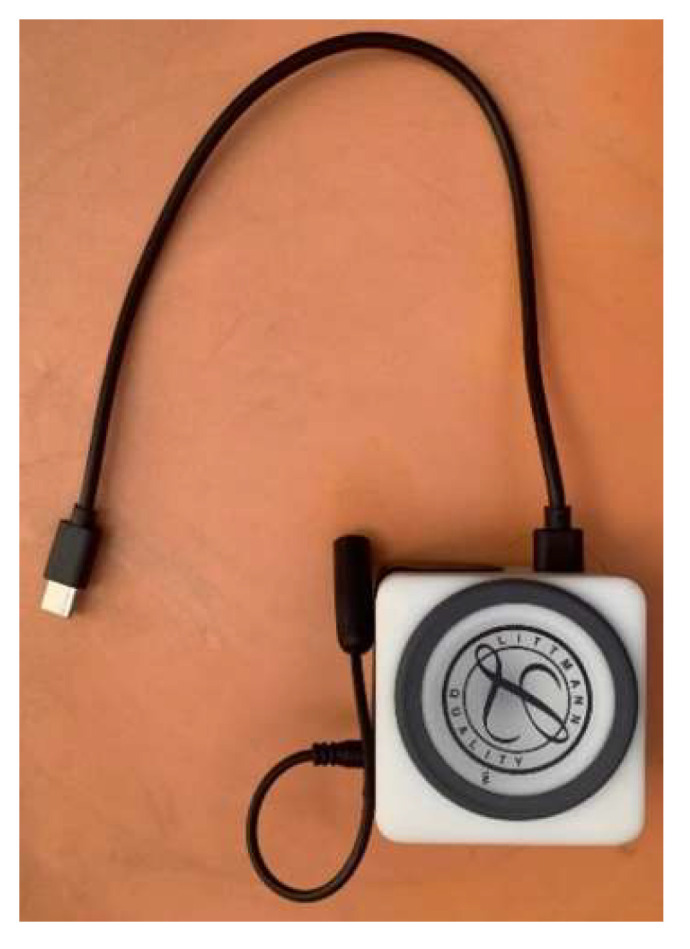
Composition of the sound acquisition device of the smart stethoscope.

We designed a digital stethoscope to acquire respiratory sounds through a diaphragm (Figure 4). The sound acquisition device consists of a sound cone, a sealed case, a stethoscope diaphragm, and a sound module. In order to make medical professionals familiar with this smart stethoscope, as a traditional acoustic stethoscope is widely used in the medical field in Thailand, we incorporated the diaphragm from a 3M^TM^ Littmann^®^ stethoscope (3M, St. Paul, MN, USA), as the stethoscope diaphragm in our device. Figure 4 and Figure 5 illustrate the assembly of the device, which transmits both digital signals through a micro USB cable and analog signals through an audio cable.

#### 3.1.2. Comparison of the Smart Stethoscope and the Two Commercial Stethoscopes: Commercial Digital Stethoscope and Traditional Acoustic Stethoscope

##### Comparison of the Smart Stethoscope and a Commercial Digital Stethoscope: Thinklabs, Wide Band, with the Measurement of the Frequency Response of Each Stethoscope Using the Power of Spectrum

**Figure 6:**
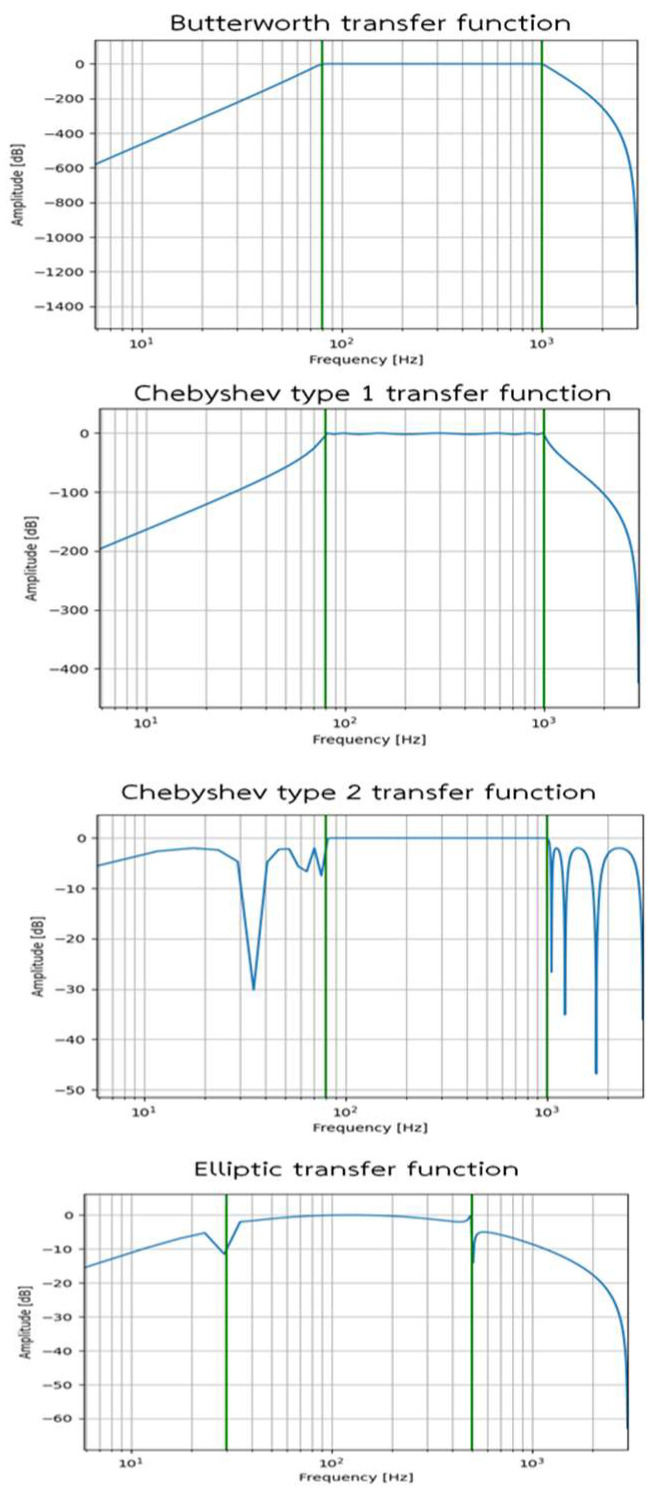
Frequency response of transfer functions: Butterworth, Chebyshev, and Elliptic.

The results of the frequency response of these filters are shown in Figure 6. The frequency response results show that the Butterworth transfer function has a stable frequency response and offers a significant difference in this bandpass. Moreover, other transfer functions showed unstable frequency responses. Therefore, it was concluded that the Butterworth filter was superior to the others, and so it was selected and employed in the system.

**Figure 7:**
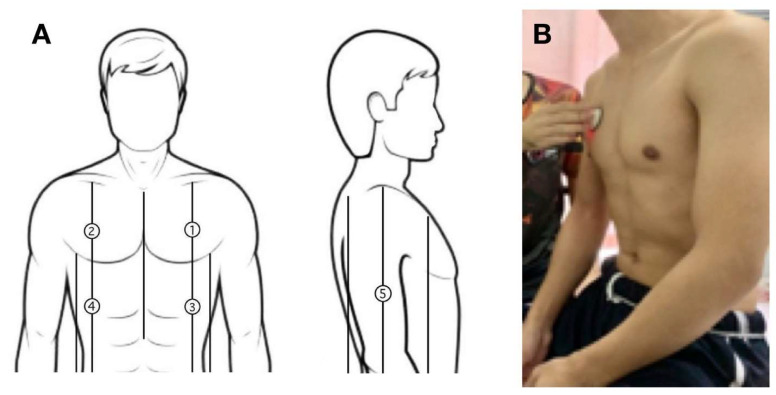
(**A**): Recording respiratory sounds at the measured position 1–5; (**B**) example of acquiring a male volunteer’s respiratory sound.

**Figure 8:**
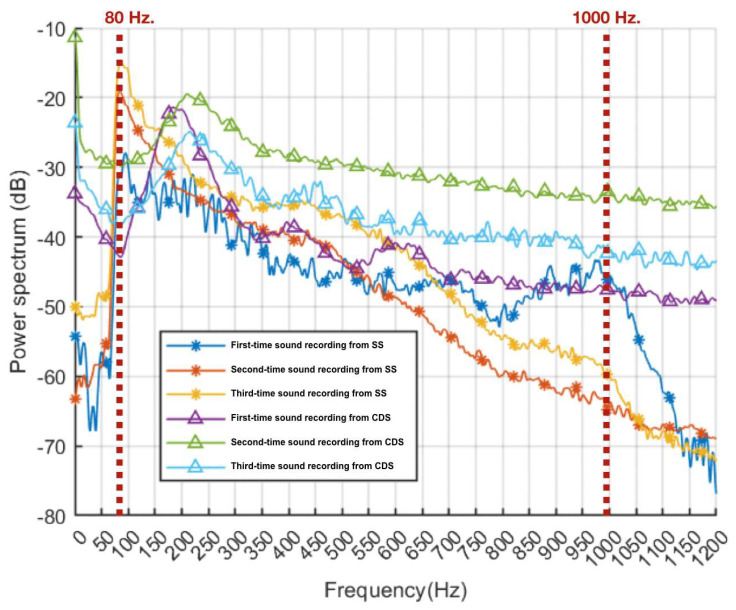
The smart stethoscope and the commercial digital stethoscope each recorded respiratory sound three times, allowing for a comparison of the power spectrum. This figure also provides insight into noise cancellation. The power spectrum of respiratory sounds recorded using the smart stethoscope was perfectly detected within 80–1000 Hz (as dashed line), and a decrement in the power spectrum was found when the sound frequency was beyond 1000 Hz, which reflected the noise cancellation ability of our smart stethoscope compared to the commercial digital stethoscope. Abbreviations: SS: smart stethoscope; CDS: commercial digital stethoscope.

To compare the performance of the smart stethoscope and a commercial digital stethoscope, respiratory sounds were collected from three volunteers at the same location (numbered 1–5), as shown in Figure 7A,B. We recorded respiratory sounds from both devices three times each to calculate their respiratory sound frequency in terms of the power spectrum. Normally, features of sound conduction in human lungs are in the 80–1000 Hz frequency ranges [37]. Therefore, Figure 8 displays the power spectrum of respiratory sounds recorded using the smart stethoscope, which were perfectly detected within 80–1000 Hz. A decrement in the power spectrum was found when the sound frequency was beyond 1000 Hz, which could reflect the noise cancellation ability of our smart stethoscope compared to the commercial digital stethoscope, which still detected respiratory sound that falls in a frequency range of more than 1000 Hz. Therefore, the smart stethoscope showed similar performance to the commercial device in the low-frequency range but showed superior performance in the high-frequency range of more than 1000 Hz, which offers insight into providing noise cancellation, especially for high-frequency noise.

##### Performance Comparison Between the Smart Stethoscope and a Traditional Acoustic Stethoscope by Professional Medical Evaluation

Performance testing was conducted to assess the efficacy and practical advantages of our smart stethoscope by comparing it to a traditional acoustic stethoscope (3M^TM^ Littmann^®^ stethoscope (3M, St. Paul, MN, USA), commonly used in clinical settings. A total of 20 medical professionals, including 9 doctors and 11 nurses from various specialties, were recruited to participate in the evaluation. All participants received instruction on how to use the smart stethoscope and its applications, which they could practice using until they became comfortable with the smart stethoscope. The performance test comparing our smart stethoscope with a traditional acoustic stethoscope began as soon as participants were well prepared. Thereafter, participants were asked to conduct a series of listening tests using both stethoscopes on simulated and live patient scenarios, enabling a comprehensive comparison of the two devices across several key performance parameters. The primary indicators assessed in this study included sound quality, loudness, noise cancellation, and the potential of each device to support advanced research applications. Sound quality focuses on the clarity and accuracy of the lung sound, which is critical for accurate diagnosis. Loudness was evaluated to determine the devices’ ability to amplify bodily sounds in noisy environments, while noise cancellation was measured for its effectiveness in reducing background noise during auscultation. Additionally, participants provided feedback on the compatibility of each stethoscope for research purposes.

**Figure 9:**
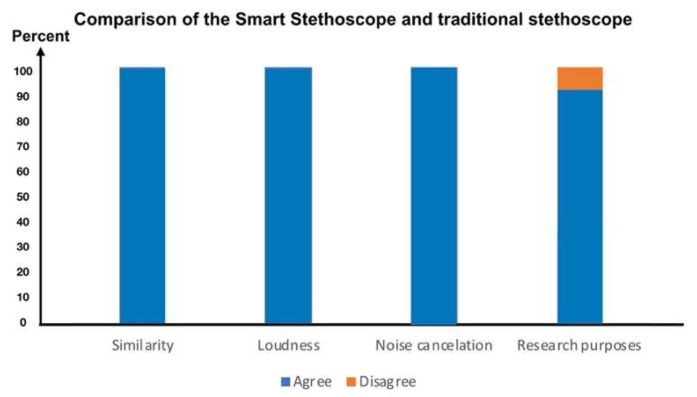
Comparison of the smart stethoscope and a traditional acoustic stethoscope (3M^TM^ Littmann^®^ stethoscope (3M, St. Paul, MN, USA) in terms of sound quality, loudness, noise cancellation, and support for advanced research applications.

As shown in Figure 9, the results revealed that all participants reported that the smart stethoscope provided sound quality comparable to the traditional acoustic stethoscope, ensuring no compromise in diagnostic accuracy (N = 20, 100%). Also, it offered similar or better loudness compared to the traditional acoustic stethoscope (N = 20, 100%), delivering clear sounds even in environments with significant background noise. The noise cancellation feature was either equal or superior (N = 20, 100%), effectively minimizing environmental disturbances and enhancing the clarity of auscultation, which could be particularly beneficial in busy hospital settings. Furthermore, the smart stethoscope demonstrated a significant advantage in supporting research applications (N = 19, 95%), with features such as digital sound recording, data storage, sharing capabilities, and potential integration with telemedicine platforms. These findings suggest that the smart stethoscope could be a valuable tool for both clinical practice and medical research.

### 3.2. Results of Mobile Application Design with Processing Server

**Figure 10:**
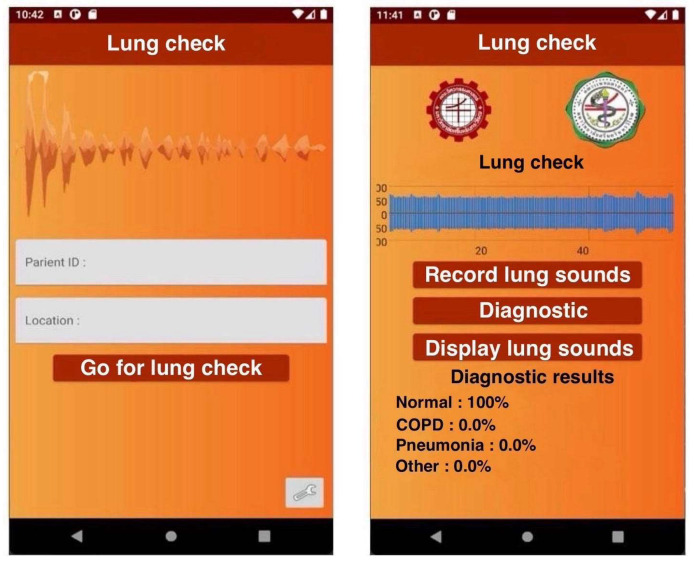
The mobile application design shows the home page, which requires the patient ID and the measurement location, and the display page, which reports the likelihood of the patient being healthy or having respiratory diseases.

The mobile application acts as an intermediary, transmitting data from the sound acquisition device for processing on the server and displaying the server’s results, as shown in Figure 10. It consists of two pages: a home page and a display page. The home page is the default page, where the patient’s ID and measurement location are entered. The display page shows the raw respiratory signal and the likelihood of the patient being healthy or having a respiratory disorder. In the experiment, pressing the record button starts recording the respiratory sound. Pressing the record button a second time stops the recording. The analysis button is then pressed to send the data to the server and retrieve the result, which is displayed on the application screen.

### 3.3. Results of Using Machine Learning Model Embedded into Cloud-Based Respiratory Disease Screening System for Real-Time Analysis

Once we developed a pre-trained ML model using Python software version 3.10, this model was embedded in the Google Cloud using the Flask web framework server. This model can classify sounds wirelessly transmitted from the mobile application to the cloud-based analysis system in real time. The system can further evaluate sound signals into four categories: healthy, COPD, pneumonia, or other respiratory diseases. The details of the system’s feature selection, accuracy, classification, and performance are shown below.

#### 3.3.1. Identification of the Best Respiratory Sound Extraction Method for Feature Selection

The Kruskal–Wallis test was applied to the extracted features to calculate the H-value. The features were ranked from high to low relevance to the class according to the H-value, as shown in Table 1. The calculated features, with an H-value ranging from 94 to 272, indicate that the features can distinctly discriminate between classes. Entropy and root mean square detail coefficients at level 6 (RMScD6) are rated as having the highest and the lowest performance associated with the class, respectively, based on a significance level of 0.05.

#### 3.3.2. Comparison of Accuracy of Selected Features

After processing with the wavelet transform and root mean square detail coefficients level 6 (RMScD6), all selected features of respiratory sound were sent to the cloud-embedded AI model for the classification of respiratory conditions. Therefore, the evaluated data from a rank of H-value were entered into the model, with 250 nodes set in the hidden layer and 1000 epochs. The feature with the highest H-value feature was calculated first. The second-highest H-value was then added to the classification model, followed by the third-highest H-value feature, and so on. The classification accuracy is shown in Table 2. It is observed that the combination of entropy, spectral entropy, and root mean square of all coefficients, except for detail coefficients at levels 1 and 6, provides the highest accuracy and the smallest difference in accuracy between the training and testing data. Therefore, it can be concluded that these features are the most suitable.

#### 3.3.3. Results of Respiratory Disease Classification and Performance of the Trained Model

**Figure 11:**
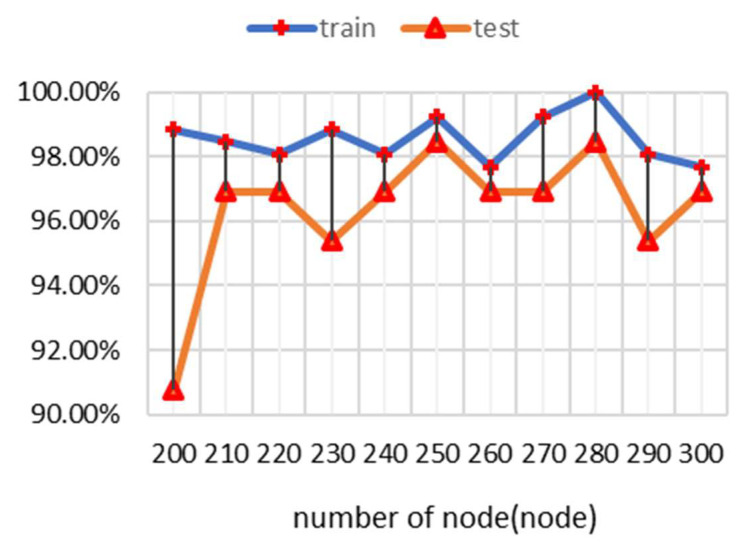
Classification accuracy from the training and test data, computed for different numbers of nodes in the hidden layer.

To find the best model for evaluating respiratory conditions, we tested the different node inputs that can offer the best performance to differentiate each respiratory condition using different numbers of nodes in the hidden layer. Using selected features from the feature selection process, selected numbers of hidden layer nodes ranging from 200 to 1000 nodes were applied to test the accuracy of the training and testing data, as shown in Figure 11. The result revealed that the model with a hidden layer of 250 nodes achieves the best test accuracy of 98% and provides the highest accuracy, with a 1% difference between training and testing accuracy. Therefore, 250 nodes is considered the optimal number of nodes.

The proposed model uses data grouped into four categories: healthy, COPD, pneumonia, and other respiratory diseases. The “Other respiratory diseases” label includes coronavirus, LRTI, URTI, asthma, bronchiolitis, and bronchiectasis. The performance of the proposed system, as shown in Table 3, yields an average sensitivity of 89.75%, specificity of 95%, and accuracy of 89%. The classification result for “Other diseases” is lower than the other categories, likely due to the complexity of combining multiple diseases.

#### 3.3.4. Results of Respiratory Disease Classification and Performance of the Tested Model with Test Data from the Database

Once the model was embedded into the server, the test data of respiratory sounds in the dataset from the database were processed and classified into four conditions. These results were further displayed on the screen. The processing results are shown in Table 4. It can be noticed that the screening results indicated that the target probability was the highest among all tests.

#### 3.3.5. Results of Respiratory Disease Classification and Performance of the Tested Model with Real-Time Data of Three Volunteers

Moreover, we tested the classification model using data recorded in real time from three healthy volunteers. We recorded respiratory sounds from five different measurement areas over the chest wall area (Figure 7A). The results showed that the real-world implementation of the tested model achieved good performance, reaching an accuracy of 80% or more in discriminating healthy volunteers from patients with pneumonia and COPD, as shown in Table 5. It is noted that the results achieved with healthy volunteers show good validity in terms of diagnosing respiratory diseases. The real-world results are relevant to the test data of respiratory signals in comparison to those obtained from the database in the test model.

## 4. Discussion

This study highlights the development of the smart stethoscope—an application-based device—to aid non-pulmonologist doctors in early screening of respiratory diseases. Several respiratory diseases, including pneumonia and chronic obstructive pulmonary disease (COPD), are prevalent and contribute to high mortality rates, particularly among the elderly [8]. However, diagnosing these conditions can be challenging for non- pulmonologist doctors, especially in rural areas or during a pandemic, when there is limited access to specialized care [42,43]. Therefore, this smart stethoscope offers valid accuracy and timely diagnosis, as the classification model can accurately identify lung sounds as normal, pneumonia, COPD, or other respiratory conditions with an impressive accuracy of 89%, sensitivity of 89.75%, and specificity of 95%. When tested on healthy volunteers, it correctly distinguished normal lungs from diseased lungs with 80% accuracy. This study underscores the potential of the smart stethoscope, particularly in resource-limited settings, as a powerful tool for the early detection and diagnosis of respiratory diseases, potentially improving patient outcomes in areas with a shortage of pulmonologists [19].

The stethoscope has been an essential tool in clinical practice for over a century, allowing doctors to listen to lung sounds and identify abnormalities associated with respiratory conditions. However, the ability to interpret these sounds effectively requires specialized training [10]. The early detection of diseases like pneumonia and COPD often hinges on subtle auscultatory findings that may be missed or misinterpreted without proper expertise [44,45]. Therefore, the smart stethoscope, as proposed in this study, could significantly enhance the diagnostic capabilities of non-pulmonologists [35]. By incorporating features such as noise cancellation and sound amplification, the device improves the quality of lung sound recordings, making it easier for clinicians to detect potential abnormalities. Moreover, the integration of machine learning models to classify lung sounds adds a layer of diagnostic accuracy that may assist in identifying conditions that are otherwise difficult to detect early. The performance testing of the smart stethoscope against commercial acoustic or digital stethoscopes yielded impressive results. Furthermore, the system’s ability to record and store lung sounds through a MEMS microphone and wirelessly transmit these data to a cloud server for real-time machine-learning analysis demonstrates the potential for remote and asynchronous diagnosis that offers potential use in telemedicine.

Our proposed machine learning model demonstrates a high accuracy of 89%, with a sensitivity of 89.75% and specificity of 95%. These results highlight the significant diagnostic potential of this technology for classifying four categories of respiratory diseases: healthy individuals, chronic obstructive pulmonary disease (COPD), pneumonia, and other respiratory diseases. Thus, it could assist medical professionals in early diagnosis. The model’s strong performance suggests that it could serve as an effective tool for the early detection and differentiation of respiratory conditions, offering valuable support to clinicians in diagnosing and managing these diseases. The sensitivity and specificity observed in our study are consistent with and comparable to those reported in several previous studies that also used computerized lung sound-based classifications for respiratory disease detection, further validating the potential of this technology [27,29,30,32].

For example, in 2019, Revathi et al. applied computerized lung sound classification to a broad spectrum of five respiratory conditions, including lower respiratory tract infections (LRTI), COPD, asthma, pneumonia, bronchiolitis, bronchiectasis, and healthy individuals [29]. Their study achieved a sensitivity of 93.74% and a specificity of 95.18%, demonstrating the robustness of lung sound-based models in identifying multiple respiratory diseases with high accuracy. Moreover, in 2021, Fraiwan et al. used a computerized lung sound classification system to detect various respiratory diseases, including healthy individuals, bronchitis, pneumonia, asthma, and heart failure [27]. Their study reported an impressive sensitivity of 95.28% and a specificity of 98.90%, demonstrating that lung sound-based models can be highly accurate in detecting a range of respiratory conditions. Similarly, in 2021, Tiwari et al. employed a lung sound-based approach to classify asthma, pneumonia, COPD, and healthy individuals [32]. They achieved a sensitivity of 72.32% and specificity of 90.66%, providing further evidence of the potential for lung sound analysis to accurately identify and differentiate between respiratory diseases. In a more recent study, in 2022, Haider et al. utilized lung sound analysis to classify COPD, asthma, and healthy individuals, achieving outstanding results with a sensitivity of 97.6% and specificity of 100% [30]. This remarkable performance highlights the potential of lung sound-based models to offer highly accurate, reliable diagnostics, even in complex cases involving conditions like COPD and asthma. Therefore, these studies collectively emphasize the effectiveness of lung sound-based models in respiratory disease detection and their potential to be refined and applied more widely in clinical practice. Our findings contribute to this growing body of evidence, suggesting that computerized lung sound analysis could play a pivotal role in the future of non-invasive, rapid, and cost-effective diagnostic tools for detection of respiratory diseases. With further development and validation, this technology could become an integral part of routine clinical assessments, particularly when access to more traditional diagnostic methods may be limited or costly, or even in pandemic or crisis situations.

However, while the results of this study are promising, several challenges and limitations should be addressed. First, although the technology demonstrated high accuracy in distinguishing between respiratory conditions, further testing is needed to evaluate its performance in a more diverse patient population and with more recruited subjects. Secondly, the smart stethoscope’s performance is highly dependent on the quality of the lung sound recordings from combined data from different respiratory sound datasets, which may contain significant data diversity from across different populations and environments, which may further affect the machine learning model’s performance. However, the model was trained on diverse data from various recording devices, enabling it to handle new data from different devices. Therefore, the proportion of data was balanced to prevent the issue of class imbalance. Additionally, we also applied normalization to minimize the impact of dataset differences. Therefore, several strategies for the next step of research are proposed: (1). increasing the amount of data for each condition and enhancing their diversity may mitigate these biases and strengthen the study; (2). combining rigorously validated and continuously updated underlying algorithms is essential to ensure their robustness in diverse clinical settings; and (3). the proposed strategies to enhance model generalizability even more, including combined-site training, federated learning, and generalization testing via transfer learning, may be applied [46]. Thirdly, the categorization of the trained model into four conditions—normal, COPD, pneumonia, and other respiratory diseases—may not provide a definite value for an accurate final diagnosis and cannot replace the expertise of pulmonologists. This is because conditions like pneumonia, COPD, and other respiratory diseases may coexist, as pneumonia can lead to COPD exacerbation or be a result of COVID-19 infection [15,16]. Additionally, COVID-19 infection can exacerbate COPD or lead to COVID-19 pneumonia [17,18]. Therefore, we envision this system as an initial tool that can help to assess the probability of the most likely respiratory conditions to support both non-MD medical professionals and non-pulmonologist doctors in providing timely, evidence-based diagnoses and aiding in treatment decisions. It can also encourage referring suspected patients to consult pulmonologists in larger hospitals. This approach not only addresses gaps in healthcare accessibility but also enhances overall patient care. Fourthly, while focusing on real-human testing in three healthy volunteers could aid in observing the impact of real-world system usage and determining the system’s efficacy in a pilot study, future studies may consider further research in a clinical setting. Moreover, respiratory signals can vary across different measurements. Therefore, using pressure signals generated by electronic systems may enhance performance and should be considered in future studies. Finally, the integration of the smart stethoscope into everyday clinical workflows may require substantial changes to medical practice. Healthcare providers, especially in rural areas, may need training not only on how to use the device but also on interpreting its results in conjunction with other clinical findings. In resource-limited settings, where access to Internet connectivity or cloud services may be intermittent, the system’s reliance on wireless transmission could pose logistical barriers. However, one of the most compelling aspects of this study is its focus on resource-limited settings, such as rural areas or in pandemic situations, where access to pulmonologists and advanced diagnostic equipment may be limited. In such contexts, the smart stethoscope could be a game-changer by enabling non-pulmonologist doctors to conduct early screening and, if necessary, refer patients for further evaluation with greater confidence. This could reduce diagnostic delays, improve treatment outcomes, and alleviate the burden on healthcare systems.

Therefore, future research should focus on refining machine learning algorithms to increase diagnostic accuracy, particularly in diverse populations and under-combining datasets, such as chest auscultation and medical imaging. Expanding the database of lung sounds used for training the system could help improve its ability to detect a broader range of respiratory diseases. Moreover, future research on using the smart stethoscope in the form of a long-term study in an actual community-based health center should be conducted; this will confirm and broaden the capability of the smart stethoscope and expand its usage not only for non-pulmonologist doctors but for all medical professionals. Additionally, incorporating feedback from healthcare providers who use the smart stethoscope in everyday practice will be essential for improving its usability, integrating it into clinical workflows, and identifying potential limitations in real-world settings.

## 5. Conclusions

This study demonstrates the promising potential of a smart stethoscope equipped with machine learning capabilities to assist non-pulmonologist doctors in the early detection of pneumonia, COPD, and other respiratory conditions. This technology could make a meaningful difference in resource-limited areas, especially during a pandemic, when access to specialized care is often delayed or unavailable, by offering a practical, affordable, and effective tool for auscultation and diagnosis.

## 6. Patent

Thai Petty Patent Certification number 23718, patent date 20 May 2024.

## Figures and Tables

**Table 1:** H-values of selected features by Kruskal–Wallis statistical test.

Feature	H-Value
Entropy before wavelet transform (Entropy)	271.246
Root mean square detail coefficients level 9 (RMScD9)	261.3169
Spectral entropy before wavelet transform (Spec.Entropy)	249.2719
Root mean square detail coefficients level 10 (RMScD10)	236.1075
Root mean square detail coefficients level 3 (RMScD3)	231.197
Root mean square detail coefficients level 2 (RMScD2)	225.9313
Root mean square detail coefficients level 4 (RMScD4)	210.049
Root mean square detail coefficients level 8 (RMScD8)	179.9965
Root mean square detail coefficients level 7 (RMScD7)	179.9965
Root mean square approximation coefficients (RMScA)	174.1438
Root mean square detail coefficients level 5 (RMScD5)	134.4328
Root mean square detail coefficients level 1 (RMScD1)	120.515
Root mean square detail coefficients level 6 (RMScD6)	94.71674

**Table 2:** Classification accuracy of the selected features.

Feature	Accuracy	
	Train	Test
Entropy	0.71	0.72
Entropy+RMScD9	0.95	0.95
Entropy+RMScD9+Spec.Entropy	0.93	0.91
Entropy+RMScD9+Spec.Entropy+ RMScD10	0.97	0.89
Entropy+RMScD9+Spec.Entropy+RMScD10+RMScD3	0.97	0.92
Entropy+RMScD9+Spec.Entropy+RMScD10+RMScD3+ RMScD2	0.97	0.94
Entropy+RMScD9+Spec.Entropy+RMScD10+RMScD3+RMScD2+ RMScD4	0.97	0.92
Entropy+RMScD9+Spec.Entropy+RMScD10+RMScD3+RMScD2+ RMScD4+RMScD8	0.98	0.95
Entropy+RMScD9+Spec.Entropy+RMScD10+RMScD3+RMScD2+RMScD4+RMScD8+RMScA	0.99	0.91
Entropy+RMScD9+Spec.Entropy+RMScD10+RMScD3+RMScD2+RMScD4+RMScD8+RMScA+RMScD5	0.99	0.98
Entropy+RMScD9+Spec.Entropy+RMScD10+RMScD3+RMScD2+RMScD4+RMScD8+RMScA+RMScD1	0.98	0.97

**Table 3:** The overall performance of the trained model.

	Sensitivity	Precision	Specificity
Healthy	0.90	0.86	0.94
Pneumonia	0.94	0.94	0.98
COPD	0.93	0.88	0.94
Other respiratory diseases	0.82	0.82	0.94
Accuracy			0.89

**Table 4:** Screening results calculated from data obtained from the test data.

Target	Screening Results Shown on the Application Page			
	Healthy	Pneumonia	COPD	Other Respiratory Diseases
Healthy	99.96%	0.0%	0.04%	0.0%
	100.0%	0.0%	0.0%	0.0%
	100.0%	0.0%	0.0%	0.0%
	100.0%	0.0%	0.0%	0.0%
	100.0%	0.0%	0.0%	0.0%
Mean (SD)	99.99% (0.02)	0.0% (0.0)	0.01% (0.02)	0.0% (0.0)
Pneumonia	0.0%	100.0%	0.0%	0.0%
	0.0%	100.0%	0.0%	0.0%
	0.0%	100.0%	0.0%	0.0%
	0.0%	100.0%	0.0%	0.0%
	0.0%	100.0%	0.0%	0.0%
Mean (SD)	0.0% (0.0)	100% (0.0)	0.0% (0.0)	0.0% (0.0)
COPD	0.0%	0.0%	92.96%	7.04%
	0.0%	0.0%	100.0%	0.0%
	0.0%	0.0%	100.0%	0.0%
	0.0%	0.0%	100.0%	0.0%
	0.0%	40.1%	59.9%	0.0%
Mean (SD)	0.0% (0.0)	8.02% (16.04)	90.57% (15.58)	1.41% (2.82)
Other respiratory diseases	0.0%	0.0%	24.45%	75.55%
	0.0%	0.0%	0.0%	100%
	0.0%	0.0%	0.0%	100%
	0.0%	0.0%	0.0%	100%
	0.0%	0.0%	0.0%	100%
Mean (SD)	0.0% (0.0)	0.0% (0.0)	4.89% (9.78)	95.1% (9.8)

**Table 5:** Example screening results recorded from three healthy volunteers. The measurements correspond to points 1 to 5, as shown in Figure 7A.

Volunteer	Screening Results Show on the Application Page			
	Healthy	Pneumonia	COPD	Other Respiratory Diseases
V1_point 1	82.53%	0.0%	0.0%	17.47%
V2_point 1	100%	0.0%	0.0%	0.0%
V3_point 1	76.1%	0.0%	0.0%	23.9%
Mean (SD)	86.01% (10.1)	0.0% (0.0)	0.0% (0.0)	13.79% (10.1)
V1_point 2	84.31%	0.0%	0.0%	15.69%
V2_point 2	100%	0.0%	0.0%	0.0%
V3_point 2	76.1%	0.0%	0.0%	23.9%
Mean (SD)	86.8% (9.91)	0.0% (0.0)	0.0% (0.0)	13.2 (9.92)
V1_point 3	72.62%	0.0%	0.0%	27.38%
V2_point 3	76.1%	0.0%	0.0%	23.9%
V3_point 3	100%	0.0%	0.0%	0.0%
Mean (SD)	82.91% (12.17)	0.0% (0.0)	0.0% (0.0)	17.1% (12.17)
V1_point 4	100%	0.0%	0.0%	0.0%
V2_point 4	85.36%	0.0%	0.0%	14.46%
V3_point 4	84.23%	0.0%	0.0%	15.77%
Mean (SD)	89.86% (7.18)	0.0% (0.0)	0.0% (0.0)	10.08% (7.15)
V1_point 5	100%	0.0%	0.0%	0.0%
V2_point 5	59.63%	0.0%	0.0%	40.37%
V3_point 5	78.88%	0.0%	0.0%	21.12%
Mean (SD)	79.5% (16.48)	0.0% (0.0)	0.0% (0.0)	20.5% (16.49)

## Data Availability

The data presented in this study are available on request from the corresponding author.
